# Violence and abuse towards general practice staff by patients and the public: a scoping review

**DOI:** 10.3399/BJGP.2024.0535

**Published:** 2025-09-23

**Authors:** Shihning Chou, Luke Sawyers, Giulia Cianci, Edward Tyrrell, Denise Kendrick

**Affiliations:** 1 School of Medicine, University of Nottingham, Nottingham, UK

**Keywords:** abuse, family medicine, general practice, primary health care, safety, workplace violence

## Abstract

**Background:**

General practice is the gatekeeper to secondary care in many countries. This unique role may expose general practice staff to violence and abuse by patients and the public, yet violence and abuse in secondary care receives more attention than that in general practice. Existing reviews on primary care do not distinguish the perpetrators of violence.

**Aim:**

To analyse the evidence on: (a) the extent, characteristics, and impact of violence and abuse by patients or the public towards general practice staff and (b) the practices relating to violence and abuse prevention and support in general practice at individual and organisational levels.

**Design and setting:**

This scoping review followed the updated Joanna Briggs Institute guidelines and the Preferred Reporting Items for Systematic reviews and Meta-Analyses Extension for Scoping Reviews guidelines.

**Method:**

Six bibliographic databases, Google, and Google Scholar were systematically searched. Deduplicated citations (*N* = 19 519) were independently screened by two reviewers. Data were extracted with a bespoke extraction form.

**Results:**

Fifty studies from 24 countries were included. Past-year rates for violence and abuse by patients and the public towards general practice staff ranged from 13.8% to 90.3% and career-long estimates were from 18.3% to 91.0%. Long waiting times and unmet patient demands were the most common reasons. It had an impact on staff mental health, turnover intention, and service capacity. No evaluated prevention or support interventions were reported.

**Conclusion:**

Violence and abuse towards general practice staff is widespread, having an impact on both individuals and service delivery. However, interventions are underresearched.

## How this fits in

Violence and abuse by patients and the public in secondary care receives more attention than that in primary care, including general practice (family medicine). This scoping review is the first, to the authors’ knowledge, to focus exclusively on patient violence and abuse towards all staff in general practice. Frequently reported reasons for abuse were long waiting times and various unmet patient demands, while commonly reported correlates with violence/abuse were female staff, younger staff, and less experienced staff members. The negative impact of violence/abuse on staff mental health, turnover intention, and service capacity has been reported in the literature, but existing research on its prevention, management, and support interventions is limited.

## Introduction

Work-related violence towards staff by patients or the public in healthcare settings is a growing concern. This constitutes one type of workplace violence, defined as *‘any act or threat of physical violence, harassment, intimidation, or other threatening disruptive behaviour that occurs at the work site. It ranges from threats and verbal abuse to physical assaults and even homicide.’* ^1^ Workplace violence can also be perpetrated by colleagues. However, findings from primary care centres,^2^ hospitals,^3^ and other pre-hospital services^4^ indicate that staff are more likely to experience violence and abuse by patients and the public than by colleagues. Therefore, this scoping review focuses on violence and abuse by patients and the public, which is simply referred to as ‘violence and abuse’ from this point on.

Primary care in the UK includes general practice (also referred to as family medicine in some countries), independent community pharmacies, community dental practices, and optical/optometry practices. GPs in the UK, like family doctors in other systems, are medical doctors who have completed specialty training and offer care alongside other professionals within their solo or group surgeries or standalone clinics and centres, separate from hospitals.^5^ Primary care services in other countries may include other medical specialties such as community paediatrics or be delivered in primary care units within a hospital. General practice or family medicine, an integral part of primary care, is the first point of contact for health care and the gatekeeper to secondary care in many countries,^6^ including the UK. This unique role may expose general practice staff to violence and abuse by patients, yet violence and abuse in secondary care or hospitals has received more attention than that in general practice. Some general practice staff are at greater risk of violence and abuse than others, with some evidence that receptionists are at particular risk.^7^ Such experiences affect staff physical and mental health, and increase levels of stress, anxiety, post-traumatic stress disorder and burnout among workers.^8^ These issues can lead to sickness absence and reduced job satisfaction.^9^ Intention to leave the job/profession is a commonly reported outcome of violence and abuse in other healthcare settings.^10^ This can put further strain on a general practice workforce that has long experienced high workload and low morale,^6^ which can in turn reduce capacity for and quality of patient care.^10^ There is an urgent need to better understand the extent and nature of violence and abuse by patients and the public in general practice settings. It is also important to explore existing research on preventive or risk-reduction measures and post-incident support in general practice.

Exploratory searches were conducted (by the second author) in 2023 to identify existing reviews and gauge the volume and nature of the literature relating to violence and abuse by patients and the public towards general practice staff. Only four relevant systematic reviews^11–14^ specifically related to general practice settings were found between 2021 and 2023, and none of the identified reviews focused on violence and abuse towards all types of general practice staff by patients and the public.

Tian *et al* ^11^ focused on workplace violence towards GPs primarily and included those who work in hospital settings without distinguishing the perpetrators of violence as either staff or patients. Pompeii *et al* ^12^ focused on outpatient clinics, which may not fully represent general practice, with the aim of including all types of workplace violence. The review by Yusoff *et al* ^13^ focused on primary care, but included both staff and patients as perpetrators. Research in other healthcare settings found that staff-on-staff abuse has greater negative consequences on the targeted individuals as well as the organisation than patient-initiated violence and abuse and requires different organisational responses.^15,16^ It would therefore be more appropriate to review these two types of workplace violence separately. In addition, the terms ‘general practice’ and ‘primary care’ have not been defined clearly or applied consistently within previous reviews and wider research, making it difficult to understand the scale of the problem and its impact in general practice. Furthermore, these systematic reviews primarily reported prevalence rates and provided little information on possible causes, correlates, impact, prevention, or support interventions. A further systematic review^14^ explored patient aggression towards GP receptionists only, excluding other staff groups. A scoping review on patient/public-to-staff violence and abuse in general practice was therefore proposed to provide an overview of evidence, with a protocol registered on Open Science Framework.^17^

The aim of this scoping review was to summarise and synthesise the evidence on:

- the extent, characteristics, and impact of violence and abuse by patients or the public towards general practice staff; and
- practices relating to violence and abuse prevention and support in general practice at individual and organisational levels.

## Method

This review followed the steps in the updated Joanna Briggs Institute methodology for scoping reviews^18^ and recommendations for the extraction, analysis, and presentation of results in scoping reviews,^19^ and are reported based on the Preferred Reporting Items for Systematic reviews and Meta-Analyses Extension for Scoping Reviews guidelines.^20^

### Eligibility criteria

#### Participants

The participants were:

- clinical and non-clinical staff;
- patients displaying/observing (from other service users) violence or abuse towards staff; and
- members of the public who accompany patients displaying/observing violence or abuse towards staff.

#### Concept

The concept was staff experiences of violence and abuse by patients and the public, direct or observed. Data were excluded if they focused on:

- staff-on-staff violence/abuse; or
- violence unrelated to work, such as crimes (for example robbery) or domestic abuse at the work site.

#### Context

The context was general practice (family medicine), or primary care settings where general practice is the core service, and the premises were dedicated to primary care services in any country. Services run as part of hospitals and standalone pharmacies were excluded.

#### Types of evidence

The types of evidence were quantitative, qualitative, or mixed methods, with no restriction on language or publication year set for the searches and citation screening.

### Search strategy

The following sources were searched systematically in September 2023 by the second author, covering citations from the date of inception of each database to the date of the searches: MEDLINE (1946 to September 2023), Embase (1974 to September 2023), Web of Science (1900 to September 2023), PsycINFO (1806 to September 2023), CINAHL (1937 to September 2023), ProQuest Dissertations & Theses (1743 to September 2023). Google and Google Scholar were searched, with the first 100 results scanned. Reference lists of all existing reviews identified during exploratory searches were scanned. Search terms included ‘violence’ or ‘abuse’ and ‘general practice’, ‘family medicine’ or ‘primary care’, and their synonyms. Both the subject headings of each database (for example, MeSH for MEDLINE) and the free text were searched. An example full syntax can be found in the registered protocol.^17^

### Citation screening

**Figure 1.**
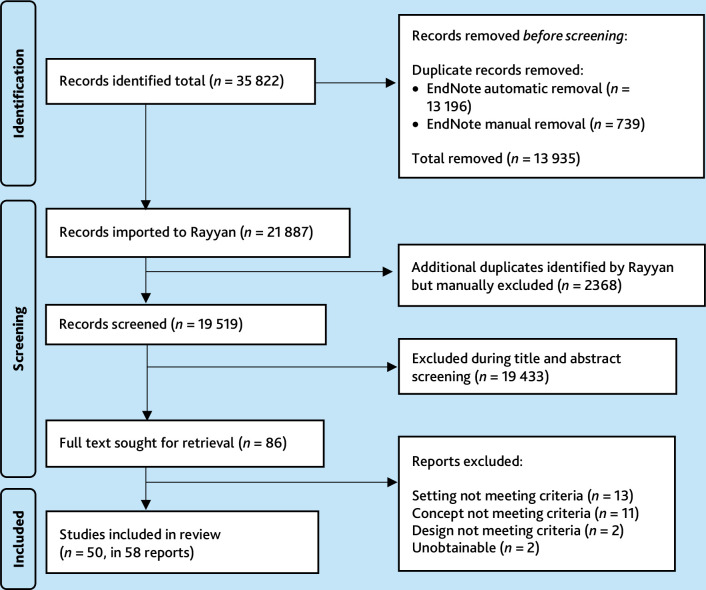
The citation screening process.

All the citations were first downloaded to Endnote for initial deduplication and then imported to Rayyan.ai (free version) for screening. The second author screened the titles and abstracts of all deduplicated citations (*N* = 19 519). The third author independently screened a random selection of 10% of the deduplicated citations. The lead reviewer (the first author) reviewed all the differences between the second and third author and made the final decision. The first and third author then screened all the relevant full texts. Any differences were resolved through discussion and agreement between the first and third author. The inclusion criteria were applied strictly where ambiguous information was excluded. Figure 1 summarises the screening process.

### Data charting and analysis

Data were extracted by the first author using a bespoke data extraction form (see the registered protocol) and checked by the third author. These included participant gender and age, study location, setting, methods, and key findings. To better understand the context that may explain the characteristics of violence and abuse, the authors also planned to extract perceived causes of violence and abuse. It was found in the initial piloting of the extraction form that some authors conflated causes with correlates. All those findings were included but further categorised into perceived causes and correlates. They are presented in separate subsections of the results below.

Following Pollack *et al*’s recommendations,^19^ the data were analysed descriptively (by the first author) by quantifying text, categorising findings based on research questions (categories reflected in subheadings), doing frequency counts, and establishing ranges. No advanced synthesis or interpretations were applied. Therefore, the findings, their wording, and explanations (or lack thereof) derived primarily from the original papers.

## Results

The full search yielded 19 519 deduplicated records. After screening titles and abstracts, two full texts were unobtainable, and 84 were screened. Of the 84 full texts, 58 were included containing 50 studies.^21–78^ Owing to the number of multiple publications for some of the studies, each study was assigned a number with all associated publications grouped together (Supplementary Table S1) and is referred to by the study number (SN) in the results section.

### Characteristics of included studies

The studies were conducted in the UK (*n* = 9), Spain (*n* = 7), Australia (*n* = 6), China (*n* = 3), Israel (*n* = 3), Norway (*n* = 3), Brazil (*n* = 2), Belgium (*n* = 2), Canada (*n* = 2), and one each in Barbados, Bosnia, Bulgaria, France, Germany, Ireland, Kuwait, Netherlands, New Zealand, Poland, Sweden, and Turkey and one in multiple countries in the West Balkans (Supplementary Table S1).

The settings included general practice (*n* = 20), primary care/community health centres (*n* = 7), family medicine practices (*n* = 4), primary care clinics or polyclinics (*n* = 5), primary care practices (*n* = 3), emergency primary care clinics (*n* = 3), community services (*n* = 1), family health units (*n* = 1), and 'Family Health Programme' (*n* = 1). Six studies simply labelled ‘primary care’ as their setting. Quantitative studies included 58 to 2940 participants whereas qualitative and mixed-methods studies included 18 to 120 participants, with a total of 23 802 across all types of studies. It is not possible to report overall gender breakdown as some studies did not report participant genders. Where figures on sexes/genders are available, 61.2% were female based on 41 studies (9094/14 863) and 38.6% were male based on 40 studies (5736/14 863). The mean response rate was 52.9% for quantitative studies. Where staff roles were identified, most were doctors (*n* = 14 280), followed by nursing staff (*n* = 2709), allied health professionals (*n* = 1876), administrative staff (*n* = 441), and receptionists (*n* = 400).

Most of the papers were in English but there was one each in the following languages: French, German, Portuguese, Spanish, and Turkish. Foreign language papers were translated using Google Translate and then checked by the third author, who is fluent in multiple European languages.

### The extent and characteristics of violence and abuse

Prevalence estimates were reported in 28 cross-sectional surveys. The most reported types of violence and abuse were verbal, physical, threats, sexual, or any type (see Table 1 for a summary and Supplementary Table S2 for details). Twenty-five studies reported percentages of staff experiencing violence and abuse in the past year and four over the career. Past-year rates for any violence and abuse ranged from 13.8% to 90.3% (14 studies), whereas career-long estimates were from 18.3% to 91.0% (four studies).

Verbal violence and abuse was most prevalent, with past-year rates of 42.1% to 89.8% (15 studies) and 87.8% for the entire career (one study). Past-year threat rates were from 6.2% to 58.5% (15 studies), and career-long rates were from 21.8%% to 59.0% (two studies). Past-year estimates of physical violence and abuse were from 0.5% to 20.6% (16 studies), with career-long rates of 17.4% to 43.8% (two studies). Past-year estimates of sexual violence and abuse were 0.1% to 22.7% (nine studies), and career-long rates were 1.1% to 10.1% (one study). Less commonly reported types included but were not limited to: defamation, slander, insults, and vexatious complaint.

**Table 1.** Rates of the most commonly reported types of violence and abuse in the past year and the entire career

Type	Past year		Career long	
	**Participants experiencing, %**	**Study number^a^**	**Participants experiencing, %**	**Study number^a^**
**Any violence/abuse**	13.8–90.3	SN2,^22^ SN6,^26^ SN8,^28^ SN13,^33^ SN16,^36^ SN19,^39–41^ SN20,^42^ SN23,^45,53,55^ SN24,^46,47^ SN25,^48^ SN26,^49^ SN27,^50^ SN34,^59^ SN36^61,62^	18.3–91.0	SN34,^59^ SN35,^60^ SN36^61,62^
**Verbal**	42.1–89.8	SN1,^21^ SN4,^24^ SN8,^28^ SN9,^29^ SN10,^30^ SN13,^33^ SN14,^34^ SN15,^35^ SN16,^36^ SN20,^42^ SN21,^43^ SN24,^46,47^ SN25,^48^ SN26,^49^ SN27^50^	87.8	SN31^56^
**Threats**	6.2–58.5	SN3,^23^ SN6,^26^ SN9,^29^ SN15,^35^ SN16,^36^ SN17,^37^ SN21,^43^ SN24,^46,47^ SN25,^48^ SN26,^49^ SN32,^57^ SN36,^61,62^ SN37^63^	21.8–59.0	SN31,^56^ SN36^61,62^
**Physical**	0.5–20.6	SN1,^21^ SN3,^23^ SN4,^24^ SN6,^26^ SN8,^28^ SN9,^29^ SN13,^33^ SN14,^34^ SN15,^35^ SN16,^36^ SN17,^37^ SN20,^42^ SN21,^43^ SN23,^45,53,55^ SN26,^49^ SN37^63^	17.4–43.8	SN31,^56^ SN36^61,62^
**Sexual**	0.1–22.7	SN1,^21^ SN8,^28^ SN9,^29^ SN14,^34^ , SN15,^35^ SN16,^36^ SN20,^42^ SN21,^43^ SN25^48^	1.1–10.1	SN31^56^
**Stalking**	2.4–13.8	SN15,^35^ SN25,^48^ SN36,^61,62^ SN37 ^63^	9.6–70.3	SN36,^61,62^
**Property damage**	3.0–31.2	SN13,^33^ SN14,^34^ SN15,^35^ SN24,^46,47^ SN25^48^	—	—

^a^Owing to the number of multiple publications for some of the studies, each study was assigned a study number (SN) with all associated publications grouped together; see Supplementary Table S1 for details. The SN is presented here together with the associated reference citation/s.

Two cross-sectional surveys (SN49,^77^ SN50)^78^ presented the figures from all of those who reported experiencing workplace violence of any kind in the past year. Out of those, 71.6% to 77.0% identified patients, their family/carers as perpetrators, and the remainder identified colleagues (11.0% to 21.0%) and leaders (9.2%).

Some studies asked participants to recall experiences in periods other than 12 months, including 78.0% of staff experiencing any violence and abuse in the past 2 years (SN11),^31^ 10.8% in the past month (SN31),^56^ and physical abuse at 0.1% and verbal abuse at 76.6% over an unspecified period (SN29).^52^ Study 18 focused on violence and abuse witnessed by practice managers rather than direct experience by individual staff members.^38^ Overall, rates of violence and abuse reported across geographical regions appeared similar.

Three studies (SN5,^25^ SN22,^44^ SN30)^54^ analysed retrospective reports of incidents over various periods rather than prevalence rates. Only two cohort studies (SN22,^44^ SN28)^51^ were found where participants reported incidents to the research team prospectively for a year, finding verbal abuse accounted for 14.5% o 31.6% of reported incidents, threats 24.7% to –33.0%, and physical aggression 30.0% to 43.7%.

Three studies provided interview data on violence and abuse, revealing that some staff faced multiple incidents and injuries (SN38,^64^ SN41,^69^ SN44).^72^ The open-text responses from a cross-sectional survey also showed that victimisation extended beyond the practice site, including *‘… humiliating text messages, complaints and published comments on internet sites or sent to other mass media, initiating petitions … filed complaints to health authorities, blackmail and extortion attempts’* (SN9).^29^

### Perceived causes/reasons for violence and abuse

The perceived causes of violence and abuse were reported in five cross-sectional surveys, two retrospective analysis of records, one cohort study, five focus group studies, three interview studies, and one of unknown qualitative methods (see Supplementary Table S2 for details).

Long waiting time was reported in nine studies – seven cross-sectional and two qualitative (SN4,^24^ SN5,^25^ SN8,^28^ SN9,^29^ SN22,^44^ SN26,^49^ SN30,^54^ SN40,^67,68^ SN44).^72^ It was unclear whether the wait was for GP consultations or hospital appointments following GPs referrals. Cross-sectional surveys reported that from 30.8% to 73.0% of participants attributed violence and abuse to long waiting times (SN4,^24^ SN9).^29^ Analyses of recorded incidents yield lower estimates, with 11.0& to 39.0% of incidents having waiting time as a cause (SN5,^25^ SN8,^28^ SN22,^44^ SN30).^54^

Other frequently reported causes were linked to a variety of unmet patient demands. Some studies did not specify what the unmet demands were (SN8,^28^ SN22,^44^ SN26,^49^ SN42,^70^ SN43,^71^ SN45),^73^ but others reported refusal to prescribe expected medications (SN5,^25^ SN27,^50^ SN30,^54^ SN39),^65,66^ dissatisfaction with the system (SN9,^29^ SN39,^65,66^ SN47),^75^ or problems with perceived treatment quality (SN9,^29^ SN30,^54^ SN46),^74^ access to care (SN46),^74^ and organisational issues (SN26,^49^ SN46)^74^ as potential causes. Failure to meet patient demands was reported in from 30.9% to 72.5% of incidents (SN8,^28^ SN22).^44^ Refusal to prescribe was reported in from 12.9% to 44.0% of incidents (SN5,^25^ SN30),^54^ with 15.2% of participants considering it a cause (SN27).^50^ Less frequently mentioned reasons included patient attitudes (SN40,^67,68^ SN46),^74^ insufficient consultation time (SN47),^75^ physical premises issues (SN9),^29^ defensive or confrontational GP measures (SN40),^67,68^ budget constraints affecting safety (SN45),^73^ organisational failure to address violence (SN40),^67,68^ and language barriers (SN46).^74^ Some studies took a view that actions of staff may have contributed to the violence experienced by suggesting possible causes such as inadequate staff training on professionalism (SN23),^45,53,55^ interpersonal skills (SN40),^67,68^ conflict management (SN47),^75^ or staff attitudes (SN9,^29^ SN26,^49^ SN46).^74^

### Possible correlates of violence and abuse

The correlates of violence and abuse were reported in 17 cross-sectional surveys, three retrospective analyses of incidents, two cohort studies, two focus groups studies, one mixed-methods study with interviews, one interview study, and one of unknown qualitative methods (see Supplementary Table S2 for details). Owing to the amount and variety of information identified, the correlates were categorised into three levels: staff, patient, and environmental.

#### Staff-level correlates

Ten studies identified staff being female as a correlate for violence and abuse overall (SN14,^34^ SN15,^35^ SN20,^42^ SN22,^44^ SN23,^45,53,55^ SN24,^46,47^ SN30,^54^ SN31,^56^ SN33,^58^ SN34),^59^ whereas two studies indicated that male staff were more likely to experience violence and abuse (SN16,^36^ SN21).^43^ Research in China found male GPs at greater risk of violence and abuse (SN16)^36^ whereas studies in Europe (SN30)^54^ and Canada (SN31)^56^ found female staff more likely to be victims. Research in Europe suggested that male staff experienced more coercion (SN30),^54^ threats, or physical attacks (SN15),^35^ whereas female staff encountered more insults (SN30).^54^ Additionally, female GPs were more likely than male GPs to encounter sexual harassment (SN15,^35^ SN31),^56^ verbal violence, and stalking (SN31).^56^ The second most reported staff-level correlates are having less work experience (SN14,^34^ SN16,^36^ SN24,^46,47^ SN33)^58^ or staff with a younger age (SN15,^35^ SN22,^44^ SN30).^54^ Findings on staff groups vary; some studies found nursing or non-medical staff, particularly receptionists or administrative staff, to be more exposed to violence and abuse than medical staff (SN22,^44^ SN45),^73^ whereas one found the opposite (SN30).^54^ Some studies found that staff on-call or working after hours (SN26,^49^ SN40)^67,68^ or home visits (SN24)^46,47^ were more likely to experience violence and abuse than those providing regular services. Minority ethnicity was identified as a correlate in a Swedish study (SN12)^32^ and a Canadian study (SN31).^56^

#### Patient-level correlates

Patient-level correlates are underresearched, with more attention to immediately noticeable factors. Substance use was identified in eight studies (SN8,^28^ SN28,^51^ SN31,^56^ SN34,^59^ SN39,^65,66^ SN40,^67,68^ SN41,^69^, SN44)^72^ and mental health difficulties were noted in seven of these (SN8,^28^ SN28,^51^ SN31,^56^, SN34,^59^ SN40,^67,68^ SN41,^69^ SN44).^72^ Male patients or members of the public (either accompanying patients or potential service users ) were more likely to initiate violent or abusive behaviours towards staff (SN5,^25^ SN11,^31^ SN22,^44^ SN31,^56^ SN34).^59^ Between 11.7% and 91.0% of participants associated substance use with violence and abuse (SN8,^28^ SN21,^43^ SN28,^51^ SN34,^59^ SN39).^65,66^ Between 32.1% and 51.0% of participants associated mental health issues with patients’ violence and abuse (SN8,^28^ SN28,^51^ SN31,^56^ SN34).^59^

#### Environmental correlates

Three studies indicated that larger practices or health centres were more exposed to such behaviours (SN7,^27^ SN18,^38^ SN45).^73^ Conversely, smaller practices were considered higher risk during after-hours care (SN40).^67,68^ One study suggested that practice layout might be associated (SN44).^72^ An Australian study found metropolitan practices more exposed to violence and abuse (SN14),^34^ whereas two studies identified rural practices as at greater risk (SN15,^35^ SN25).^48^ Additionally, one study found that practices in more socioeconomically deprived areas were more exposed to violence and abuse (SN24).^46,47^

### Impact of violence and abuse

The impact of violence and abuse was reported in 16 cross-sectional surveys, three retrospective analyses of incidents, one cohort study, one mixed-methods study with interviews, three interview studies, and one focus groups study (see Box 1 for a summary and see Supplementary Table S2 for details). Owing to the amount and variety of information identified, the authors further categorised the impact into three: individual level, service/organisational level, and coping responses (Box 1).

**Box 1.** Impact of violence and abuse^a^

Individual level	Service/organisational level	Coping responses
Stress, distress, trauma, anxiety (SN15,^35^ SN22,^44^ SN39,^65,66^ SN41,^69^ SN44,^72^ SN48)^76^ Changes in overall functioning or wellbeing (SN4,^24^ SN5,^25^ SN11,^31^ SN41)^69^ Depressive symptoms (SN6)^26^ Changes in personal relationships (SN36)^61,62^ Fear and apprehension (SN3,^23^ SN24,^46,47^ SN36,^61,62^ SN39,^65,66^ SN41,^69^ SN42)^70^ Reduced emotional wellbeing (SN8,^28^ SN23)^45,53,55^ Reduced physical wellbeing (SN14)^34^ Physical injury (SN44)^72^	Avoiding certain times, locations, or patients (SN5,^25^ SN44)^72^ De-registering more patients (who behave in an abusive/violent manner) (SN19)^39–41^ Reduced job satisfaction (SN23,^45,53,55^ SN41)^69^ Reduced confidence and commitment (SN19)^39–41^ Turnover intention (SN12,^32^ SN41,^69^ SN44)^72^ Reduced performance (SN41)^69^ Reduced service capacity (SN14)^34^ Staff taken off duty (SN22)^44^ Sick leave (SN33)^58^	Increased security measures, including the use of panic buttons (SN19,^39–41^ SN44)^72^ Change daily or work routine (SN36,^61,62^ SN43)^71^ Seeking violence prevention courses (SN43)^71^ Reflecting on dynamics in consultation and planning for similar situations in the future (SN44)^72^

^a^Owing to the number of multiple publications for some of the studies, each study was assigned a study number (SN) with all associated publications grouped together; see Supplementary Table S1 for details. The SN is presented here together with the associated reference citation/s.

Violence and abuse negatively affected staff mental health, particularly stress and anxiety (one cross-sectional study, one cohort study, and four qualitative studies). Only one study (qualitative) mentioned physical injury. Organisational consequences included staff being taken off duty or taking leave (two cross-sectional studies), avoiding certain times, locations, or patients (three studies), intending to leave their job or profession (three studies), and reduced job satisfaction (three studies), potentially having an impact on performance (one study) and reduced service capacity (one study). Some staff installed panic buttons (three studies) or changed routines and practices (two studies) following violence and abuse.

### Suggested prevention strategies

Prevention strategies and their feasibility were less commonly reported (see Box 2 for a summary and Supplementary Table S3 for details). Only three cross-sectional surveys were found, two retrospective analyses of incidents, one cohort study, two focus groups studies, one mixed-methods study with interviews, and one qualitative study with unspecified methods. Nevertheless, the information identified was varied and therefore further categorised into two areas: practice provisions and staff attitude, knowledge, and conduct (Box 2). Overall, only one qualitative study on intervention development was found (SN45).^73^ No studies were found evaluating interventions to prevent or manage violence and abuse. Nonetheless, 10 studies documented current (SN3,^23^ SN13,^33^ SN22,^44^ SN36)^61,62^ and suggested (SN5,^25^ SN30,^54^ SN43,^71^ SN44,^72^ SN45,^73^ SN47)^75^ measures. This analysis does not distinguish between pre- and post-incident support or intervention.

**Box 2.** Suggested and current incident-specific measures^a^

Practice provisions	Attitude, knowledge, conduct
Staff training on incident management and violence prevention (SN3,^23^ SN13,^33^ SN30,^54^ SN43)^71^ Reducing caseloads and increasing consultation time (SN13)^33^ Talking to the patient (SN22)^44^ Providing patients with information on the role of the service (SN43)^71^ Sending letter to the patient about their behaviour (SN36)^61,62^ Assigning another professional to the patient (SN36)^61,62^ Reporting to authority (SN5)^25^ Defining recording system and encouraging recording (SN45)^73^ Develop and review model responses and protocol (SN45,^73^ SN47)^75^ Team approach to designing model/system/responses (SN45)^73^ Joint examination of impact (SN45)^73^ Providing alarms or peer check-ins (SN45)^73^ Improving practce routines and services (SN43)^71^ Supportive management and follow-up (SN43)^71^	Move to private area as an issue arises and talk things through with patient (SN45)^73^ More information to team about particular symptoms/patients (SN45)^73^ ‘Time out’ for staff (SN45)^73^ ‘No blame’ discussion of incidents (SN45)^73^ Encourage staff not to condone or tolerate incidents (SN45)^73^ Collective rather than individual responsibility (SN45)^73^ Non-judgemental analysis (SN45)^73^ Reduce avoidance mechanisms in future (SN45)^73^

^a^Owing to the number of multiple publications for some of the studies, each study was assigned an SN with all associated publications grouped together; see Supplementary Table S1 for details. The SN is presented here together with the associated reference citation/s. SN = study number.

At the staff level, training on incident management (SN3,^23^ SN13,^33^ SN30,^54^ SN43)^71^ was most frequently recommended. Other suggestions on training (content details not provided) included self-defence (SN13),^33^ violence prevention (SN13),^33^ critical incident review, communication skills with role play, learning minority languages, and staff mentoring (SN45).^73^ At the service level, reducing caseloads and increasing consultation time were proposed to alleviate staff–patient tensions (SN13).^33^ Regarding incident-specific measures, two main areas were discussed: practice provisions and staff/management attitude, knowledge, and conduct (see Box 2). The recommended practices included fair and supportive responses with a non-judgemental, collaborative approach, without condoning poor behaviours. However, the authors did not elaborate who those practices were aimed at or the content to be implemented.

Police involvement was rarely suggested. Some staff felt reluctant because of fear of reprisal, aversion to added paperwork, and the belief that publicising violence would encourage further incidents (SN44).^72^

## Discussion

### Summary

This scoping review investigated the extent, characteristics, and impact of violence and abuse, and the practices relating to violence and abuse prevention and support at individual and organisational levels. The search identified 50 relevant studies conducted worldwide. Violence and abuse is widespread, with past-year rates between 13.8% and 90.3% and lifetime rates from 18.3% to 91.0%. Experiences vary widely, including verbal, physical, sexual abuse, threats, stalking, and property damage, with verbal abuse being most common. Incidents occur both on-site and online. The most common reasons for violence and abuse in general practice include long waiting times and unmet patient expectations. Staff and patient attitudes were noted but underresearched. Research has focused mainly on doctors, with administrative staff the least studied. Less experienced or younger staff may experience more violence and abuse. Female staff are more likely to experience violence and abuse. Male patients and those with substance use and mental health needs are more commonly associated with violence and abuse. It has an impact on individual staff as well as general practices as it increases staff stress, anxiety, depression, and trauma, and in turn affects service delivery. While prior research involved participants in developing potential interventions, no studies evaluating the effectiveness of these proposed solutions were found.

### Strengths and limitations

This review focused mainly on published literature (with the exception of dissertations and theses, and the first 100 results from searches of Google and Google Scholar) from English-language databases using a range of search terms to identify violence and abuse (violen* or abus* or aggress* or threat* or harass* or assault), even though the languages were not restricted in the searches. Searching more grey literature sources and foreign-language databases along with adding search terms such as intimidation may have resulted in additional relevant studies being included in this review.

Studies varied in their definitions of general practice and primary care, which may have affected the accuracy of citation screening because of insufficiently clear definitions. Most of the studies used a cross-sectional design with self-report questionnaires or a qualitative approach with interviews or focus groups. Self-reported data may be subject to recall and measurement bias, limiting understanding of the true extent of violence and abuse and its impact. Although only two studies reported the association between victimisation experience and minoritised ethnicity of the staff, which may indicate the role of racism, the true extent of this association may be underestimated as survey participants from minoritised communities often decline to declare their ethnic identity.^30^ Analyses of official records may be more reliable, but they suffer from selection bias. The dark figure of unreported workplace violence in hospitals is estimated to be 88% and only serious incidents are formally reported.^79^ Furthermore, the amount of detail provided in interventional studies was insufficient for further application. Regarding the population, most of the research focused on GPs, with administrative and reception staff receiving the least attention. The COVID-19 pandemic has had long lasting negative effects on access to health care and more recent studies may find different prevalence rates.

### Comparison with existing literature

This scoping review included more studies than Pompeii *et al*’s systematic review in 2023^12^ on workplace violence against outpatient clinic staff. The extent of violence and abuse found in the current review is greater than in Pompeii *et al*’s review except for verbal abuse. The extent may be greater than other healthcare settings. The proportion of participants experiencing violence and abuse in most UK surveys^30,31,39,41^ including the current review is over 60%, while 25.5% of staff report experiencing bullying, harassment, and abuse from service users in the NHS Staff Survey in which general practice is not included.^80^ The impact found in this scoping review aligns with that across healthcare settings, except financial consequences. However, it is not possible to compare the severity of violence and abuse nor the seriousness of its impact as they have not been measured in research. It is difficult to compare the perceived causes and correlates to wider literature as they did not distinguish staff or patients as perpetrators. Although training on violence prevention is commonly recommended across settings, some of the suggestions are hospital oriented and do not apply to general practice, such as having dedicated security personnel.^81^

### Implications for research and practice

This scoping review has identified a substantial body of literature to enable a systematic review of the prevalence of and risk factors for violence and abuse towards general practice staff. Such a review should include a thorough exploration of definitions of violence and abuse used and explore a wide range of risk factors at environmental (such as geographical, rural/urban, deprivation), organisational (such as healthcare settings, systems, and cultures), and individual levels (such as staff and perpetrator characteristics). Separate systematic reviews on the correlates and perceived causes of violence and abuse, and of interventions to prevent violence and abuse, would also be useful. Future reviews should consider grey literature and foreign database searching, and explore the use of a wider range of search terms describing violence and abuse.

More studies are needed exploring violence and abuse towards general practice staff post-COVID and as the use of digital technology to access GP services increases. In addition, the intersection between violence and abuse and wider societal factors (for example, deprivation) requires further study. Primary qualitative studies exploring explanations for the varied experiences of different professional groups and genders, and the role played by the prevailing societal culture (such as gender roles, stereotyping), would be helpful. With the increased use of online technology, it is also important to explore digital and cyber abuse.

Owing to the nature and fundamental purpose of scoping reviews, policy and practice implications can only be discussed tentatively. Nevertheless, the high prevalence of violence and abuse and its negative impact on individuals and services indicate the need for effective preventive strategies, staff support, and addressing organisational factors contributing to violence and abuse.

In conclusion, this review focused on violence and abuse excluding staff-on-staff abuse. It specifically examined general practice and settings where general practice was the primary service, excluding hospital-based services because measures or strategies developed for other contexts may not apply to general practice. The study’s findings go beyond those from previous systematic reviews, which did not differentiate between violence and abuse by patients and the public and violence from colleagues, and identify what additional research is required.
